# Quantitative Radiomics: Impact of Pulse Sequence Parameter Selection on MRI-Based Textural Features of the Brain

**DOI:** 10.1155/2018/1729071

**Published:** 2018-07-30

**Authors:** John Ford, Nesrin Dogan, Lori Young, Fei Yang

**Affiliations:** ^1^Department of Radiation Oncology, University of Miami Miller School of Medicine, Miami, FL 33136, USA; ^2^Department of Radiation Oncology, University of Washington, Seattle, WA 98195, USA

## Abstract

**Objectives:**

Radiomic features extracted from diverse MRI modalities have been investigated regarding their predictive and/or prognostic value in a variety of cancers. With the aid of a 3D realistic digital MRI phantom of the brain, the aim of this study was to examine the impact of pulse sequence parameter selection on MRI-based textural parameters of the brain.

**Methods:**

MR images of the employed digital phantom were realized with *SimuBloch*, a simulation package made for fast generation of image sequences based on the Bloch equations. Pulse sequences being investigated consisted of spin echo (SE), gradient echo (GRE), spoiled gradient echo (SP-GRE), inversion recovery spin echo (IR-SE), and inversion recovery gradient echo (IR-GRE). Twenty-nine radiomic textural features related, respectively, to gray-level intensity histograms (GLIH), cooccurrence matrices (GLCOM), zone size matrices (GLZSM), and neighborhood difference matrices (GLNDM) were evaluated for the obtained MR realizations, and differences were identified.

**Results:**

It was found that radiomic features vary considerably among images generated by the five different T1-weighted pulse sequences, and the deviations from those measured on the T1 map vary among features, from a few percent to over 100%. Radiomic features extracted from T1-weighted spin-echo images with TR varying from 360 ms to 620 ms and TE = 3.4 ms showed coefficients of variation (CV) up to 45%, while up to 70%, for T2-weighted spin-echo images with TE varying over the range 60–120 ms and TR = 6400 ms.

**Conclusion:**

Variability of radiologic textural appearance on MR realizations with respect to the choice of pulse sequence and imaging parameters is feature-dependent and can be substantial. It calls for caution in employing MRI-derived radiomic features especially when pooling imaging data from multiple institutions with intention of correlating with clinical endpoints.

## 1. Introduction

Given the noninvasive nature of medical imaging along with its ready availability, radiomic features, that is, radiographic cancer imaging traits, have recently been sought after actively as prognostic and/or predictive indicators under the hypothesis that tumoral radiologic appearance conveys underlying phenotypic and/or genetic diversity [1–6]. Multifarious radiologic features ranging from statistical parameters of intensity histogram to spatial interactions between intensity levels to textural heterogeneity measures and to morphological descriptor have been brought up in relation to this context for imaging modalities including computed tomography (CT), magnetic resonance imaging (MRI), ultrasound, and positron emission tomography (PET) amongst others in a variety of cancers [7–9].

As for MRI, radiomic features have been assessed in diverse clinical sites such as the brain, head and neck, breast, kidney, bladder, prostate, and extremities [8, 10–18]. The results of these studies demonstrate that radiomic features may hold potential for patient stratification and subsequent treatment adaptation. However, successful translation of radiomics research into clinic will greatly depend on the repeatability, reproducibility, and validity of the radiomic features being investigated. By repeatability, it means that given the same subject and imaging protocol, the same results will consistently occur while reproducibility is a measure of consistency from one scanner or one institution to another. Validity however relates to the extent to which radiomic features measure the underlying construct they purport to measure [19]. These concerns have been investigated by several studies with emphasis being laid, respectively, on the effects of radiomic features due to MRI field strength, imaging protocols, and manufacturers through the use of either living subjects and/or physical phantoms [20–25]. One of the major shortcomings of these studies was the lack of ground truth information of radiomic features of the objects of interest. In the absence of absolute knowledge of the radiomic features of an object, repeatability and reproducibility of radiomic features with respect to MR imaging parameters can be assessed only to a limited degree, and the critical issue of validity yet remains unanswered. With the aid of a realistic 3D digital MR phantom of the human brain, the objective of the current study was to examine the impact of pulse sequence parameter selection on MRI-based textural features of the brain.

## 2. Materials and Methods

### 2.1. MRI Simulation

**Figure 1:**
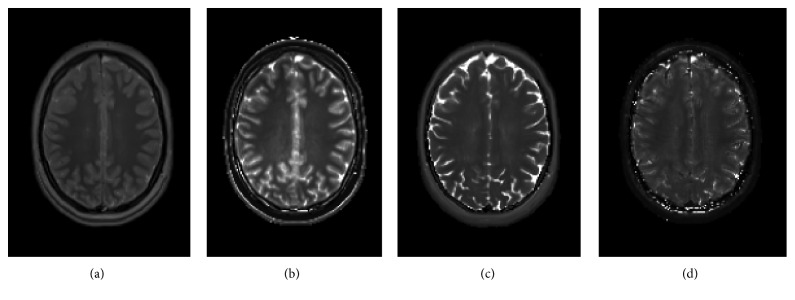
Representative slice of quantitative maps comprising the 3D digital phantom: (a) *M*_0_, (b) *T*_1_, (c) *T*_2_, and (d) *T*_2_^*∗*^.

A realistic digital phantom serving as the source of generating MR images was adopted. The utilization of a digital phantom has the principal advantage of the existence of a known ground truth for the assorted radiomic parameters. For each voxel of the digital phantom, nuclear magnetic resonance (NMR) properties including equilibrium magnetization (*M*_0_), longitudinal relaxation time (*T*_1_), transverse relaxation time (*T*_2_), and transverse relaxation time with extra dephasing effects (*T*_2_^*∗*^) were defined [26].  Figure 1 shows one representative axial slice of the *M*_0_, *T*_1_, *T*_2_, and *T*_2_^*∗*^ maps comprising the digital phantom. MR images of the employed digital phantom were realized using *SimuBloch*, a simulation package implemented for fast production of image sequences based on solving the Bloch equations, which is hosted on the Virtual Imaging Platform (VIP), a computing platform providing high computational resources for multimodality medical image simulation [27]. Pulse sequences being considered consisted of spin echo (SE), gradient echo (GRE), spoiled gradient echo (SP-GRE), inversion recovery spin echo (IR-SE), and inversion recovery gradient echo (IR-GRE).

To investigate the reliability of radiomic feature with respect to pulse sequence choice, T1-weighted images of the 3D phantom were generated using the following parameters: for SE, TR/TE = 500/8.4 ms; for GRE, TR/TE = 120/8.0 ms; for SP-GRE, TR/TE = 35/6.0 ms and FA = 40°; for IR-SE, TR/TE/TI = 2400/20.0/1200 ms; and for IR-GRE, TR/TE/TI = 1900/2.98/900 ms. To investigate the dependence of radiomic features on TR and TE in T1-weighted spin echo, MR images were generated with TR varying from 360 ms to 620 ms in increments of 10 ms and TE ranging from 3.4 ms to 13.4 ms in increments of 0.5 ms. Finally, to investigate the dependence of radiomic features on TE in T2-weighted spin echo, MR images with TR = 6400 ms and TE varying from 60 ms to 120 ms were generated. All of the aforementioned pulse sequence parameters were chosen to reflect T1 and T2 weightings commonly used for 3T clinical brain MRI [28].

### 2.2. Radiomic Analysis

**Figure 2:**
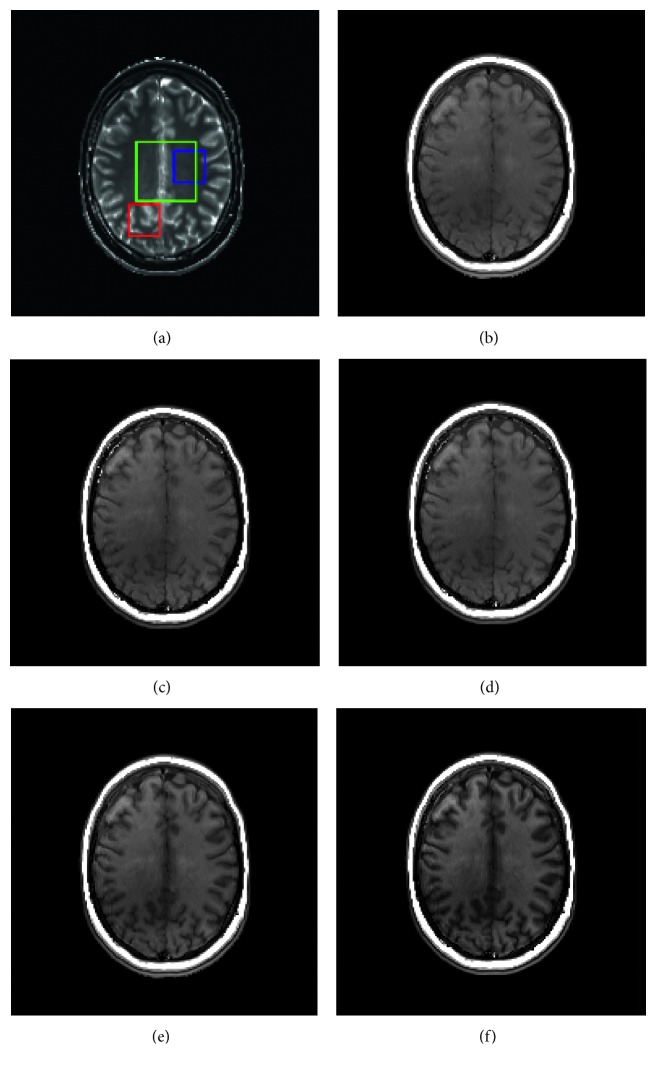
(a) Central slice of the volumes of interest (VOI) being utilized, overlaid on the T1 map of the digital phantom, with VOI 1 in blue, VOI 2 in red, and VOI 3 in green. T1-weighted images generated from (b) spin echo (SE), (c) gradient echo (GRE), (d) spoiled gradient echo (SP-GRE), (e) inversion recovery spin echo (IR-SE), and (f) inversion recovery gradient-echo (IR-GRE).

Radiomic textural analysis for different MR realizations was performed within three cubical volumes of interest (VOIs) with one featuring less signal heterogeneity (VOI 1), a second associated with relatively strong signal heterogeneity on either the *T*_1_ map and/or the *T*_2_ map (VOI 2), and a third larger volume encompassing both types of regions (VOI 3). The VOIs specified are visualized in Figure 2(a). Prior to feature extraction, intensity values within the considered VOIs were linearly rescaled to the range of integers [0, 255]. The reason that intensity normalization [29, 30] was not considered lies in the fact that MR data of the present study were derived from simulations and impacts of scanner-dependent variations on the image data would be minimal at best, if not virtually nonexistent. Radiomic parameters being assessed consisted of an array of frequently referenced textural features derived, respectively, from gray-level intensity histograms (GLIH), gray-level cooccurrence matrices (GLCOM), gray-level neighborhood difference matrices (GLNDM), and gray-level zone size matrices (GLZSM) [9, 31–33]. The considered GLIH-based features included variance, skewness, and kurtosis with each characterizing the shape of intensity histogram, respectively, from the aspect of dispersion, symmetry, and peakedness. For GLCOM, with a voxel displacement of 1, the neighboring properties of the voxels in the 13 directions of 3D space were taken into account simultaneously by adding up the intensity cooccurrence patterns into one single matrix [8]. GLCOM-based textural features capture local spatial properties of the image via examination of the joint occurrence probability of one gray-level value relative to another at a specified linear displacement. GLNDM-based features, with a neighborhood size of 3 × 3 × 3, exploit visual perceptual property of textures by discerning the spatial details within an image in terms of the gray-level difference between image voxels and their local neighborhoods. GLZSM-based features depict regional spatial properties of the image content by taking account of the spatial frequency of contiguous regions that encompass voxels sharing identical gray-level values. Radiomic analysis was carried out using an in-house developed program [34, 35], and summarized in Table 1 are the imaging features being investigated. Radiomic metrics extracted from the resultant MR images were compared to identify the influence on textural metrics due to the pulse sequence selection and pulse sequence parameter variation.

### 2.3. Statistical Analysis

Statistical analysis was performed using JMP Pro® (Version 13, SAS Institute Inc., Cary, NC). Textural features measured in images generated from the 3D digital phantom using the five T1-weighted pulse sequences were compared to those measured in the T1 map, by calculating the absolute percentage error. Coefficient of variation (CV) was calculated for spin-echo T1-weighted images with varying TR and T2-weighted images with varying TE.

## 3. Results

Examples of axial slices of T1-weighted images generated from the 3D digital brain phantom utilizing various pulse sequences are shown in Figure 2 with panel (b) for spin echo (SE), (c) for gradient echo (GRE), (d) for spoiled gradient echo (SP-GRE), (e) for inversion recovery spin-echo (IR-SE), and (f) for inversion recovery gradient-echo (IR-GRE). As can be seen from the figure, the simulated images, though qualitatively similar, manifest different degrees of T1 weighting.

**Figure 3:**
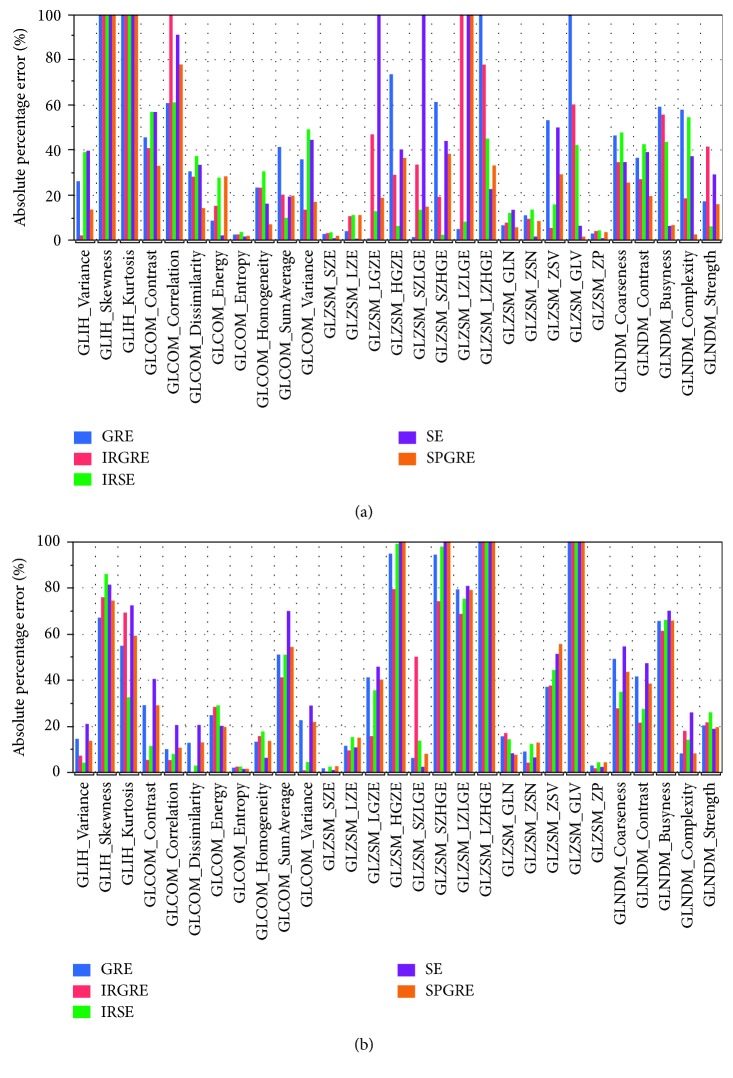
Absolute percentage error for textural features from T1-weighted images shown in Figure 2 and measured in (a) VOI 1, a relatively homogeneous region, and (b) VOI 2, a more heterogeneous region of the brain in relative to those measured from the T1 map.

Figure 3(a) shows a comparison of textural features measured in VOI 1 in each T1-weighted image in relation to those measured from the T1 map. VOI 1 featured a relatively homogeneous region in the T1 maps and would be expected to bear less relevance to nontrivial spatial variation in terms of T1-weighted signal intensity; however, the majority of the textural metrics derived from the resultant T1-weighted images yielded substantial differences from those extracted from the T1 map. Considerable differences in textural parameters were observed also between each T1-weighted image and the T1 map for VOI 2, as is presented in Figure 3(b). A comparison of textural parameter variation patterns between VOI 1 and VOI 2 reveals that, for regions with relatively homogeneous signals, GLIH-based features have the most radical changes while GLZSM-based features for those regions with heterogeneous signals. With GLIH-based metrics depicting global spatial attributes, GLZSM-based depicting regional spatial attributes, and GLCOM-based and GLNDM-based features depicting local spatial attributes, these results demonstrate that there exist extensive amounts of spatial intensity alteration across diverse scales between the images and the map data. From Figure 3, it can also be readily appreciated that different pulse sequences, though all accentuating T1 weighting, capture and reflect the actual spatial characteristics of the T1 map to greatly differing degrees.

**Figure 4:**
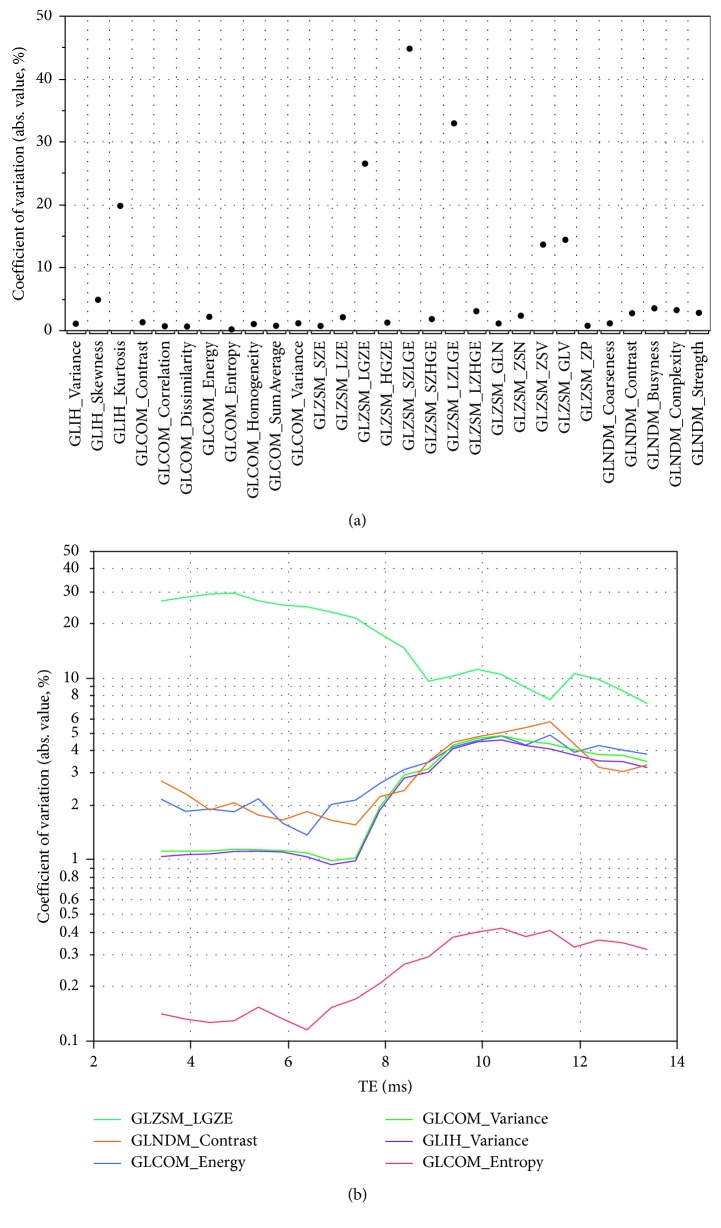
(a) Coefficient of variation (CV) for textural features in VOI 3 for T1-weighted spin-echo images with TR varying over the range 360–620 ms and shortest TE (TE = 3.4 ms). (b) Dependence of several textural features' CV on TE for T1-weighted spin-echo images with TR varying over the range 360–620 ms, showing the effect of admixing T2 weighting into the nominally T1-weighted image.

Dependence of radiomic features on pulse sequence parameters (i.e., TR and TE) was assessed for T1-weighted spin-echo imaging. MR images of spin echo with TR varying from 360 ms to 620 ms in increments of 10 ms and TE ranging from 3.4 ms to 13.4 ms in increments of 0.5 ms were generated.  Figure 4(a) shows CV of each radiomic feature measured in VOI 3 over the range of TR being examined with TE being fixed to 3.4 ms, the shortest one investigated. While for most of the features CVs are less than 5%, it is noted that several, including GLIH-based kurtosis, GLZSM-based LGZE, SZLGE, LZLGE, ZSV, and GLV have much larger variability with CV in the range 15–45%. Although there is no clear threshold of CV for acceptable reproducibility of MRI-based radiomic parameters, greater CVs would imply these features are associated with poor, if not lacking, values towards clinical relevance. The impact of TE selection on radiomic parameter variability was examined too, and Figure 4(b) shows the effect on the variability of several example features measured in VOI 3 when using increased TE values in T1-weighted spin-echo imaging with TR ranging from 360 ms to 620. It can be seen that, for the presented radiomic feature, the selection of TE may exercise considerable influence on the variation being due to varying TR.

**Figure 5:**
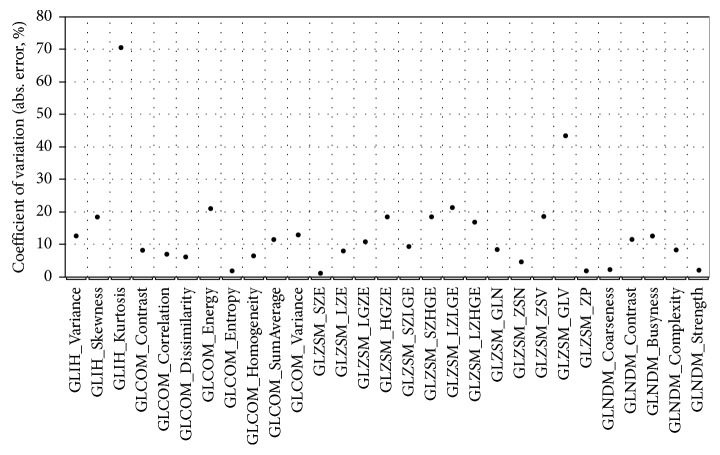
Coefficient of variation (CV) for textural features in VOI 3 for T2-weighted spin-echo images with TE varying over the range 60–120 ms and TR = 6400 ms.

Dependence of radiomic parameters on TE in T2-weighted spin-echo imaging was explored aided by generation of MR images of spin echo with TR of 6400 ms and TE varying from 60 ms to 120 ms in increments of 10 ms.  Figure 5 shows the CV of each radiomic feature measured in VOI 3 for the generated T2-weighted images. Most of the radiomic features exhibit CV less than 25%, except for GLIH-based kurtosis and GLZSM-based GLV which assumed, respectively, a value of 70% and 43%. In comparison to those shown in Figure 4 for T1-weighed imaging, radiomic textural features, in general, demonstrate greater variability with respect to T2-weighted imaging.

## 4. Discussion

Radiomics seeks to build image-based predictive risk models for diagnosis and prognosis. In the case of tumor diseases, such models may predict, for example, grade, stage, response, recurrence free survival, overall survival, and radiation-induced toxicity, based on imaging modality. Given that a great number of radiomic features, also known as image biomarkers, may be extracted, the imminent question arose as to which features are relevant and reproducible. There are, in general, two main approaches to tackle this question. The first one is the agnostic approach. In this approach, predictive models are built using large heterogeneous image data for a certain outcome and specific tumor phenotype with all image features entering the radiomics process followed by subsequent screening for robustness and relevancy. The advantage of this approach is that it is relatively straightforward and more closely approaches the end goal, that is, the building of clinical models for treatment individualization. The disadvantages are that the reason why a feature is incorporated in the model, and how a feature is directly related to tumor phenotype and/or genotype, is obscured. The amount of image data required for such an approach is voluminous which makes data accrual and retrieval extremely difficult for any given single center, if not impossible.

The second approach is prescriptive and is intended to seek out robust radiomic features based on reproducibility analysis and relevant biomarkers using a univariate correlation with clinical end points of interest. This approach can reveal the reason why a feature is being selected in the model. However, in this approach, there are many parameters involved throughout the whole process for radiomics analysis, ranging from imaging acquisition to preprocessing steps, and to feature extraction, and consequently, there are many degrees of freedom to deal with. The impetus for this work was to determine the impact of some key MR imaging parameters on radiomic features of the brain; however, it is not feasible to pick images in databases to achieve uniformity of imaging parameters, as doing so would severely diminish the usable number of data. We, therefore, turned to simulation to allow us to explore a large MR acquisition space and the variance of radiomic features in that large space.

The important results reported in this study may be summarized as follows: (1) radiomic textural features vary considerably on T1-weighted images produced by different pulse sequences; (2) radiomic textural features on T1-weighted images can deviate considerably from those in the T1 maps; (3) for spin-echo imaging, textural features can depend strongly on choice of TR and TE; and (4) errors and variances mentioned above are feature dependent.

It is perhaps not surprising that different T1-weighted pulse sequences produce images in which the textural features vary since we did not attempt to normalize the degree of T1 weighting. What is somewhat surprising is the degree to which pulse sequence choice impacts many textural features and moreover, that there is such disparity for some features among images that appear very similar. Furthermore, it is striking how much some features on these T1-weighted images differ from the T1 map. The clinical significance of these findings, we believe, lies in the utilization of T1- and T2-weighted images as input to any predictive risk models of brain disease. To the extent that certain features are more or less robust to MRI acquisition details, they may be more or less weighted as input to risk model, resulting in a more accurate prediction. Most, if not all, models in use today are agnostic regarding radiomic features and their weightings in the development of models [6]. Also, to the extent that the models' predictive power depends on biophysical properties of the tissue related to T1 or T2, which manifest themselves in T1- or T2-weighted MR images, radiomic features that capture the true T1 or T2 (i.e., features with small error) will provide a stronger and more reliable input. The variability of features seen across pulse sequences led us to focus on the TR/TE dependence within one pulse sequence (spin echo). The CVs for spin echo are in general greater for T2- versus T1-weighted images; the significance of this observation is yet to be determined but again, may be important when weighting image data used as input to predictive risk model. It is interesting to see the dependence of CV on TE in T1-weighted images. This is presumably due to admixture of T2 weighting to a nominally T1-weighted image. Although ideally one would use the minimum TE to maximize signal-to noise, it may be necessary to increase TE to accommodate, for example, flow-compensated gradient pulses, or lower bandwidth for higher spatial resolution. Again, we see that it is important to recognize that TE may influence the fidelity of T1-weighted images.

Early work on application of texture analysis to MR images of gel and polystyrene sphere phantoms indicated sensitivity of texture features to MR acquisition details including TR, TE, and bandwidth, but robustness of texture pattern discrimination as long as the spatial resolution was sufficiently high [22, 23]. A multi-institutional study of 73 subjects found that texture analysis applied to T1- and T2-weighted brain images using three different MR scanners was able to discriminate tissue type (white/gray matter, CSF, and tumor) despite nonuniform acquisition protocols [21]. A study of the effect of MR slice thickness in texture-based classification of normal tissue versus plaque in multiple sclerosis patients showed only moderate differences between 1 mm and 3 mm slices [24]. Likewise, a phantom study using clinical breast imaging protocols showed that acquisition parameters may not greatly influence the ability of texture analysis to differentiate different texture phantoms [17]. The essence of this prior work is that while some texture features may be sensitive to MR acquisition details, there are others that are able to correctly classify tissue independent of MR acquisition details. Our results are in agreement with the prior work, in that we found that some texture features are stable across the clinically relevant range of TR/TE. The advantage of our simulation approach is that we are able to explore the stability of features over a wide array of MR acquisition protocols. Another advantage of our simulation approach is that we are able to compare MRI-based texture features with the ground truth. We have shown that there can be very large absolute errors for many features for some pulse sequences. The degree to which this difference from ground truth affects the ability of texture analysis to capture tissue heterogeneity/composition, which is known to be important in characterizing tumor aggressiveness, is unclear at this point.

Although this work is illuminating and is to our knowledge the first attempt to determine the radiomic feature dependence on MRI acquisition details using comparison to the underlying map data, there are several limitations. First, we examined normal brain images only. Future work will utilize quantitative images of disease, as MRI acquisition dependence of diseased tissue radiomic features, and/or those of organs at risk during treatment, is of supreme clinical importance. Secondly, our chosen VOIs did not correspond to any particular anatomy. VOI 1 and VOI 2 were chosen as they are visually distinct regions, one being mostly homogeneous and the other with obviously more heterogeneity; VOI 3 is a slightly larger region encompassing both, and all VOIs were roughly on the order of the size of a typical glioma. Further work will incorporate expertly delineated regions corresponding to relevant normal brain structures as well as pathology. Furthermore, in this work, we focused solely on the influence of pulse sequence, TR, and TE. Future work will incorporate into the MRI image generation the effects of noise, field strength, reconstruction algorithm, image artifacts, and other MR acquisition techniques including diffusion-weighted imaging to better mimic image generation in the clinic.

## 5. Conclusion

Radiomic features vary considerably among images generated by the five different T1-weighted pulse sequences, and a great number of the features deviate considerably from those measured on the T1 map. For the spin-echo pulse sequence, a certain number of the features measured on nominally T1- and T2-weighted images depend strongly on choice of TR/TE, even with TR/TE restricted to a range normally encountered in clinical MRI. The clear implication is that there exist sources of variability that can confound studies, especially those pooling imaging data from multiple institutions, attempting to link MRI-derived radiomic features to biomarkers and clinical outcomes.

## Figures and Tables

**Table 1:** MRI radiomic textural features being examined.

Category	Feature
Based on gray-level intensity histogram (GLIH)	Variance
	Skewness
	Kurtosis

Based on gray-level cooccurrence matrix (GLCOM)	Contrast
	Correlation
	Dissimilarity
	Energy
	Entropy
	Homogeneity
	SumAverage
	Variance

Based on gray-level zone size matrix (GLZSM)	Short zones emphasis (SZE)
	Large zones emphasis (LZE)
	Low gray-level zones emphasis (LGZE)
	High gray-level zones emphasis (HGZE)
	Short zones low gray-level emphasis (SZLGE)
	Short zones high gray-level emphasis (SZHGE)
	Large zones low gray-level emphasis (LZLGE)
	Large zones high gray-level emphasis (LZHGE)
	Gray-level nonuniformity (GLN)
	Zone size nonuniformity (ZSN)
	Zone size variance (ZSV)
	Gray-level variance (GLV)
	Zone percentage (ZP)

Based on gray-level neighbourhood difference matrix (GLNDM)	Coarseness
	Contrast
	Busyness
	Complexity
	Strength

## Data Availability

The data that support the findings of this study are available from the corresponding author upon reasonable request.
